# Supplementary material for: Metabolomic Analysis Reveals the Mechanisms of Hepatotoxicity Induced by Aflatoxin M1 and Ochratoxin A
Source: Toxins (Basel). 2022 Feb 15;14(2):141. doi: 10.3390/toxins14020141 (PMC8880135; doi:10.3390/toxins14020141)
Supplement: Supplementary file 1 [file toxins-14-00141-s001.zip › toxins-1507589-supplementary.pdf]

A **Control vs AFM1**

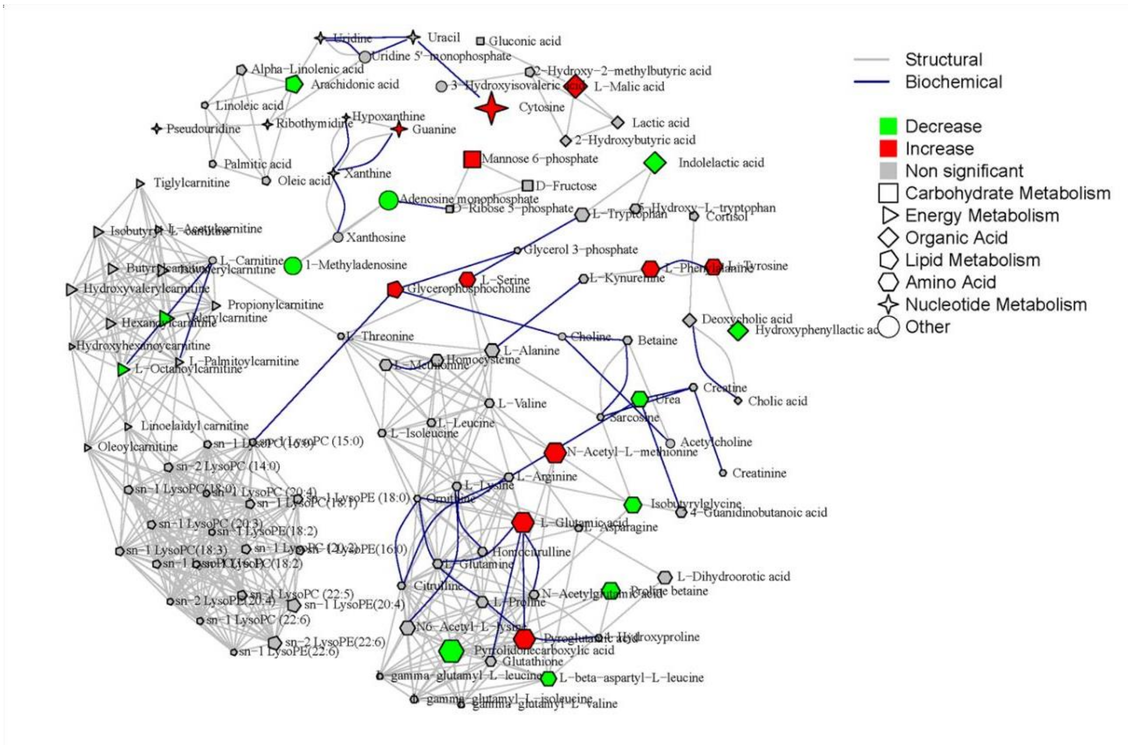

B **Control vs OTA**

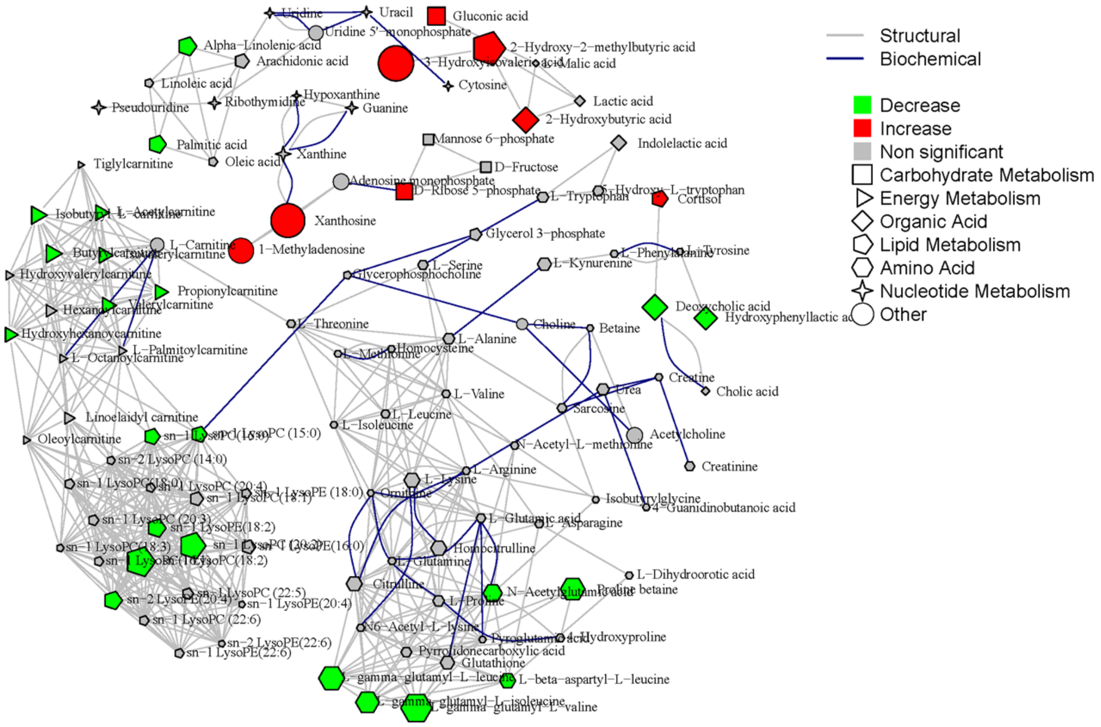

C Control vs AFM1+OTA

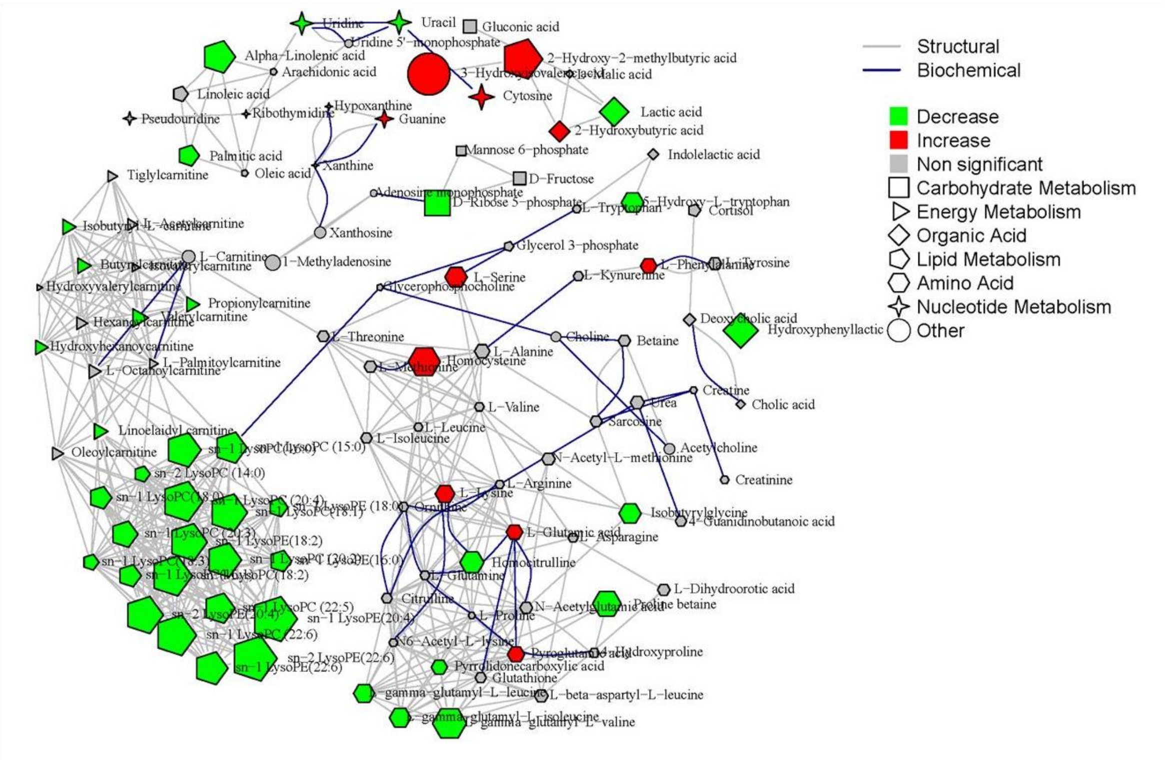

D

	AFM1	OTA	AFM1+OTA
Up regulated	11	8	11
Down regulated	12	23	41
No change	119	111	90

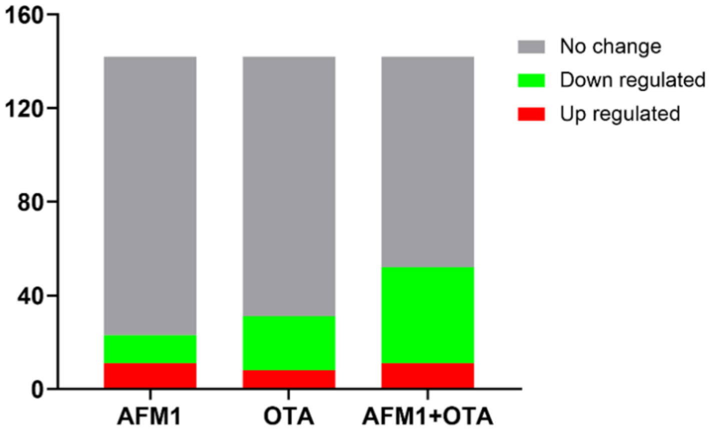

Figure S1. Metabolite integrated metabolic pathway analysis in liver of mice treated with individual and combined AFM1 and OTA. (A) Control vs AFM1, (B) Control vs OTA, (C) Control vs AFM1+OTA and (D) the number of changed metabolites in different mycotoxins treatment. Red represents the upregulated metabolites, and the green represents the downregulated metabolites with criterion of FC > 1.50, and p < 0.05.

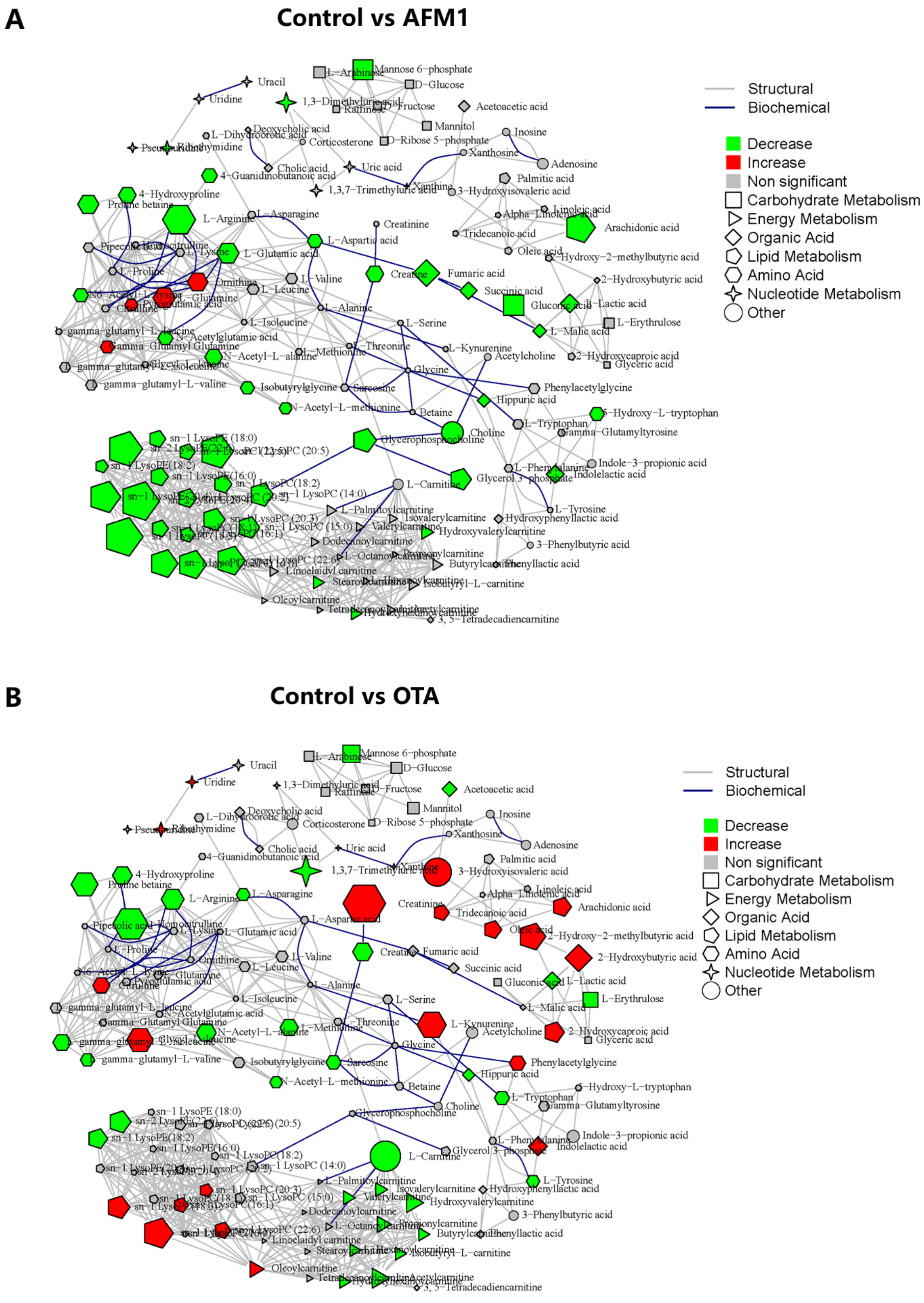
A
Control vs AFM1
Structural
Biochemical
Decrease
Increase
Non significant
Carbohydrate Metabolism
Energy Metabolism
Organic Acid
Lipid Metabolism
Amino Acid
Nucleotide Metabolism
Other
B
Control vs OTA
Structural
Biochemical
Decrease
Increase
Non significant
Carbohydrate Metabolism
Energy Metabolism
Organic Acid
Lipid Metabolism
Amino Acid
Nucleotide Metabolism
Other

C **Control vs AFM1+OTA**

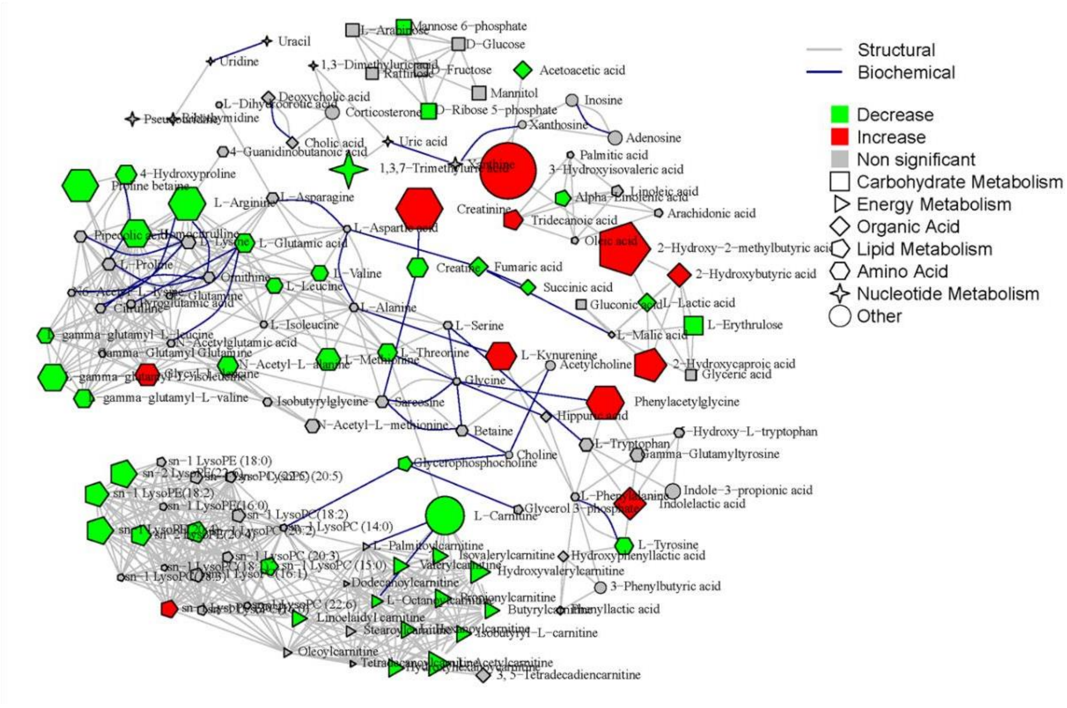

D

	AFM1	OTA	AFM1+OTA
Up regulated	4	19	11
Down regulated	49	32	43
No change	116	118	115

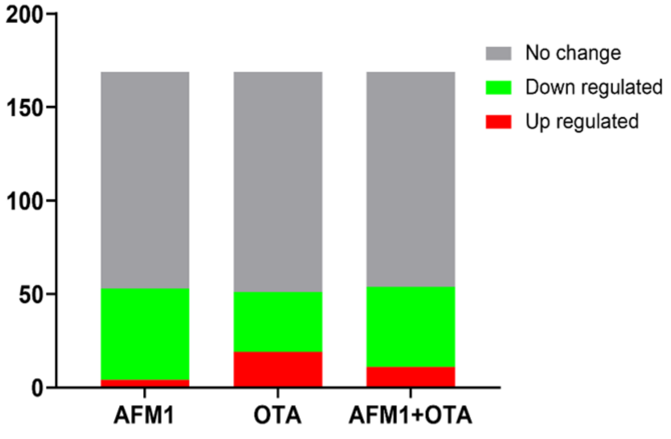

Figure S2. Metabolite integrated metabolic pathway analysis in serum of mice treated with individual and combined AFM1 and OTA. (A) Control vs AFM1, (B) Control vs OTA, (C) Control vs AFM1+OTA and (D) the number of changed metabolites in different mycotoxins treatment. Red represents the upregulated metabolites, and the green represents the downregulated metabolites with criterion of FC > 1.50, and p < 0.05.

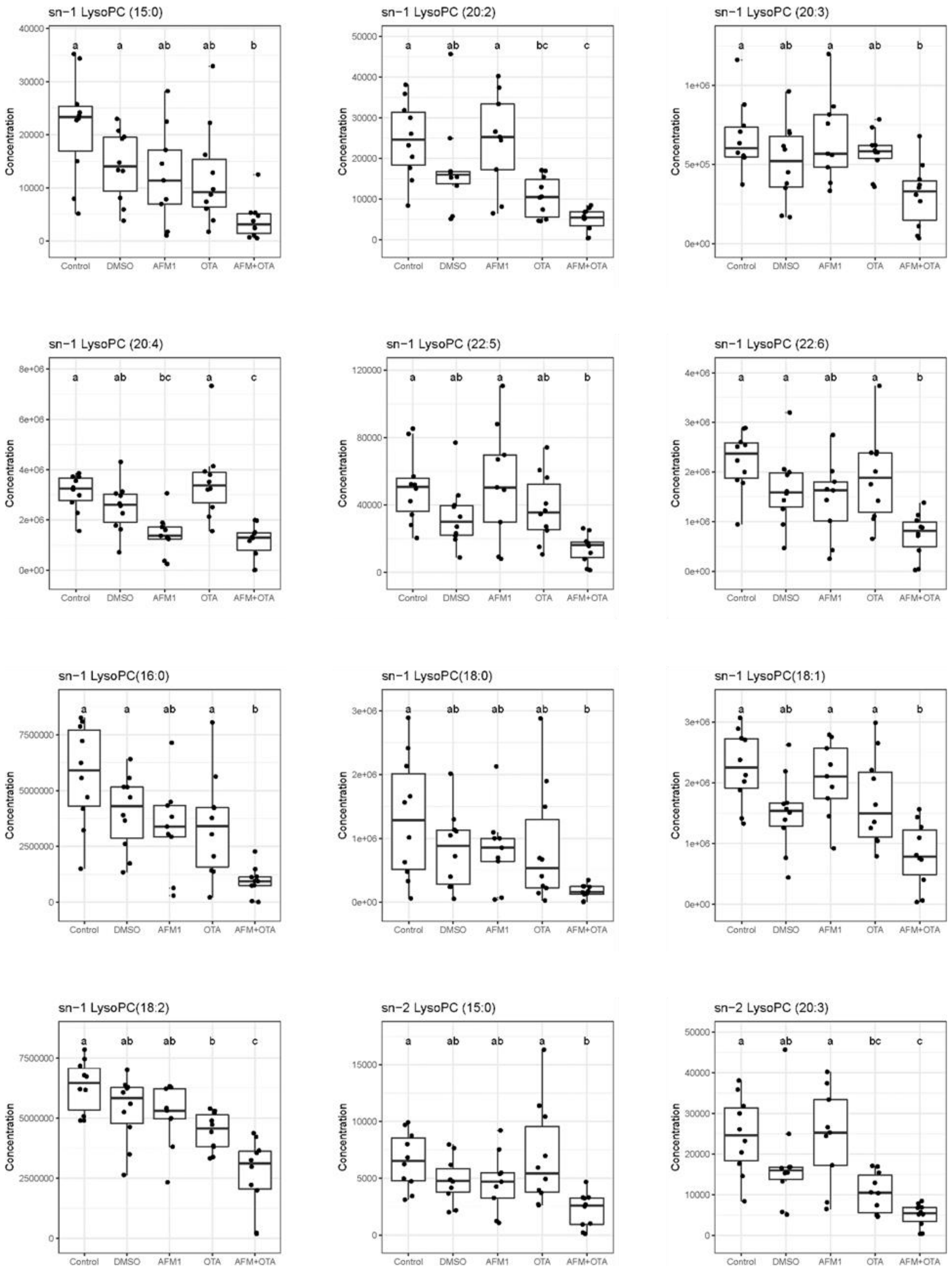
sn−1 LysoPC (15:0)
a a ab ab b
sn−1 LysoPC (20:2)
a ab a bc c
sn−1 LysoPC (20:3)
a ab a ab b
sn−1 LysoPC (20:4)
a ab bc a c
sn−1 LysoPC (22:5)
a ab a ab b
sn−1 LysoPC (22:6)
a a ab a b
sn−1 LysoPC(16:0)
a a ab a b
sn−1 LysoPC(18:0)
a ab ab ab b
sn−1 LysoPC(18:1)
a ab a a b
sn−1 LysoPC(18:2)
a ab ab b c
sn−2 LysoPC (15:0)
a ab ab a b
sn−2 LysoPC (20:3)
a ab a bc c
Concentration
Control
DMSO
AFM1
OTA
AFM+OTA

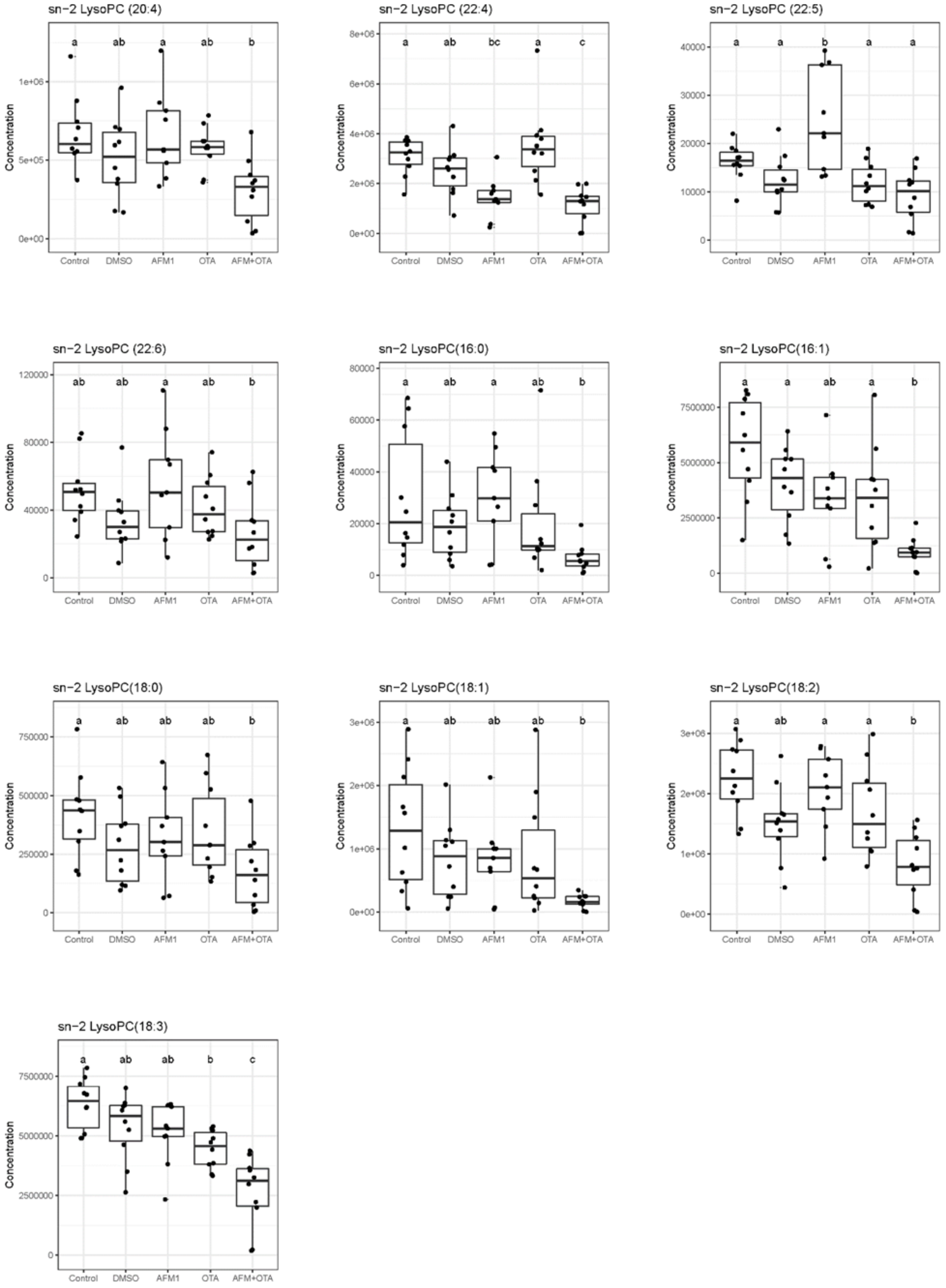

Figure S3. The concentration of significant changed LysoPCs in liver.

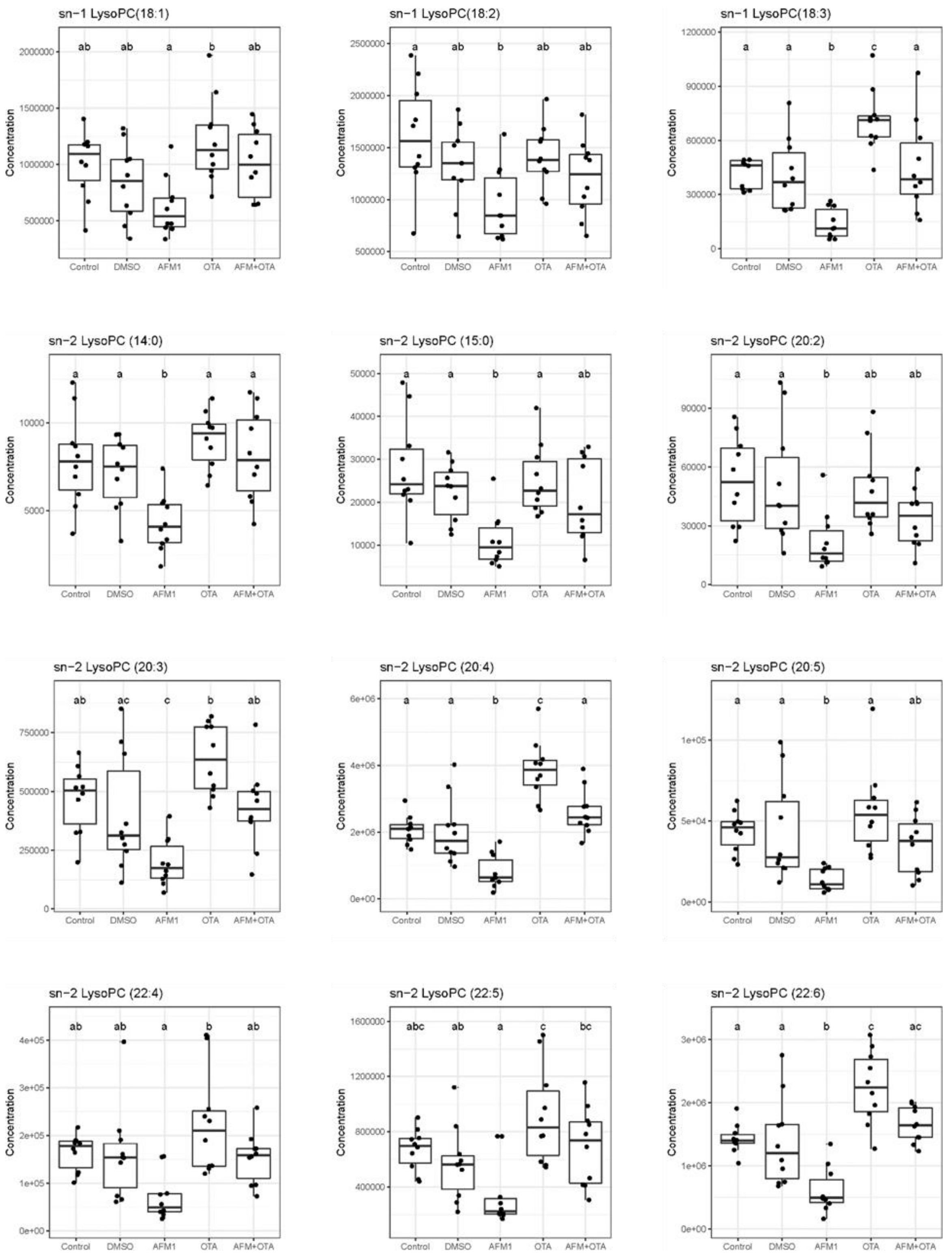
sn−1 LysoPC(18:1)
ab ab a b ab
2000000
1500000
1000000
500000
Concentration
Control DMSO AFM1 OTA AFM+OTA
sn−1 LysoPC(18:2)
a ab b ab ab
2500000
2000000
1500000
1000000
500000
Concentration
Control DMSO AFM1 OTA AFM+OTA
sn−1 LysoPC(18:3)
a a b c a
1200000
900000
600000
300000
0
Concentration
Control DMSO AFM1 OTA AFM+OTA
sn−2 LysoPC (14:0)
a a b a a
10000
5000
Concentration
Control DMSO AFM1 OTA AFM+OTA
sn−2 LysoPC (15:0)
a a b a ab
50000
40000
30000
20000
10000
Concentration
Control DMSO AFM1 OTA AFM+OTA
sn−2 LysoPC (20:2)
a a b ab ab
90000
60000
30000
0
Concentration
Control DMSO AFM1 OTA AFM+OTA
sn−2 LysoPC (20:3)
ab ac c b ab
750000
500000
250000
0
Concentration
Control DMSO AFM1 OTA AFM+OTA
sn−2 LysoPC (20:4)
a a b c a
6e+06
4e+06
2e+06
0e+00
Concentration
Control DMSO AFM1 OTA AFM+OTA
sn−2 LysoPC (20:5)
a a b a ab
1e+05
5e+04
0e+00
Concentration
Control DMSO AFM1 OTA AFM+OTA
sn−2 LysoPC (22:4)
ab ab a b ab
4e+05
3e+05
2e+05
1e+05
0e+00
Concentration
Control DMSO AFM1 OTA AFM+OTA
sn−2 LysoPC (22:5)
abc ab a c bc
1600000
1200000
800000
400000
Concentration
Control DMSO AFM1 OTA AFM+OTA
sn−2 LysoPC (22:6)
a a b c ac
3e+06
2e+06
1e+06
0e+00
Concentration
Control DMSO AFM1 OTA AFM+OTA

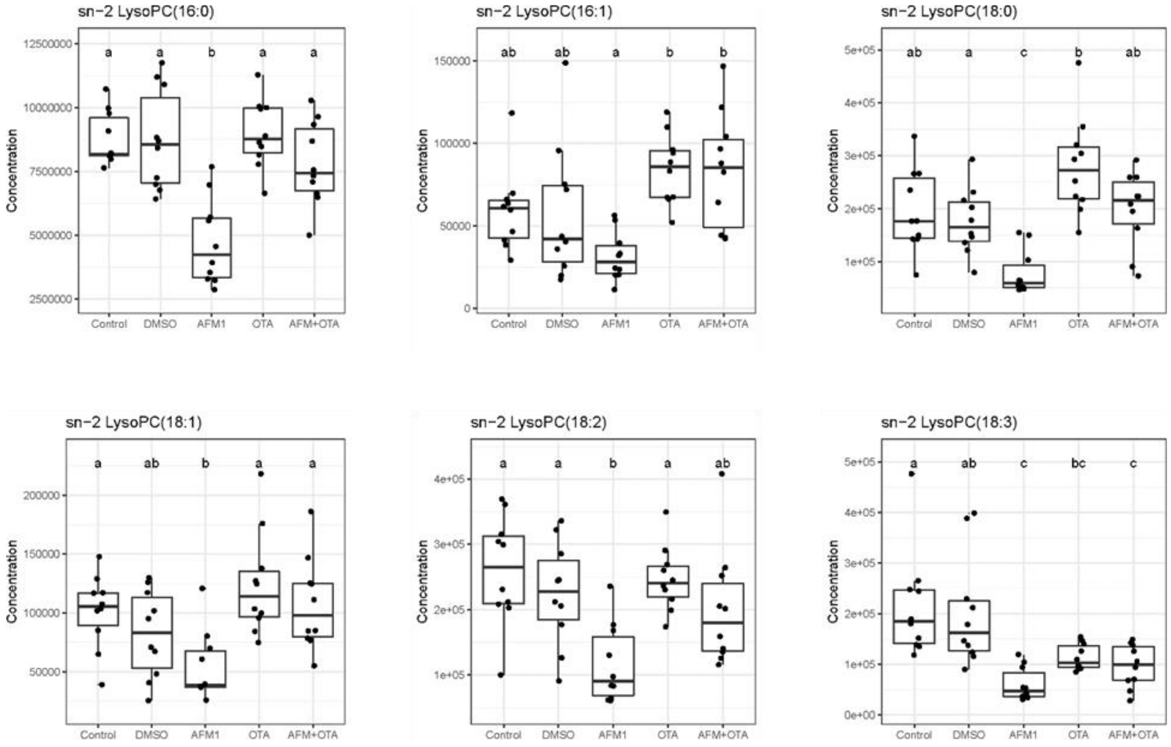

Figure S4. The concentration of significant changed LysoPCs in serum.

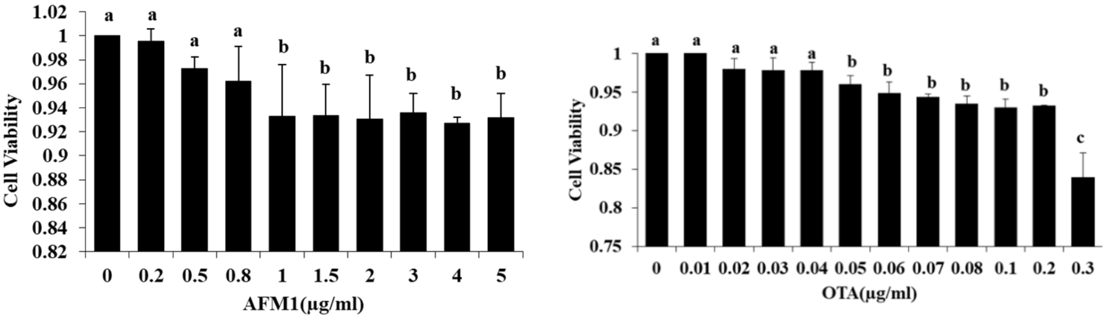

Figure S5. Cytotoxic effects induced by AFM1 and OTA on HepG2 cells.

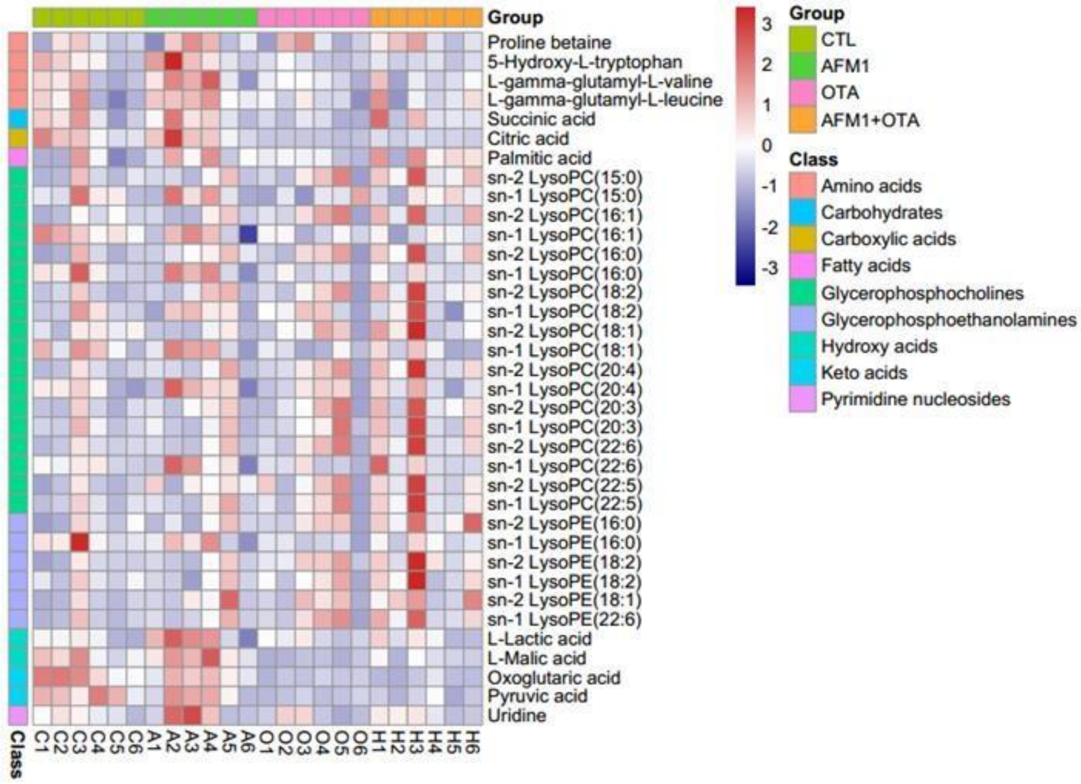

Figure S6. Heat map representation of metabolites in the HepG2 cell under different conditions of mycotoxins treatment. Red and blue represent higher and lower level of the respective metabolites. C represents control group, A represents individual AFM1 group, O represents OTA group and H represents AFM1+OTA group.

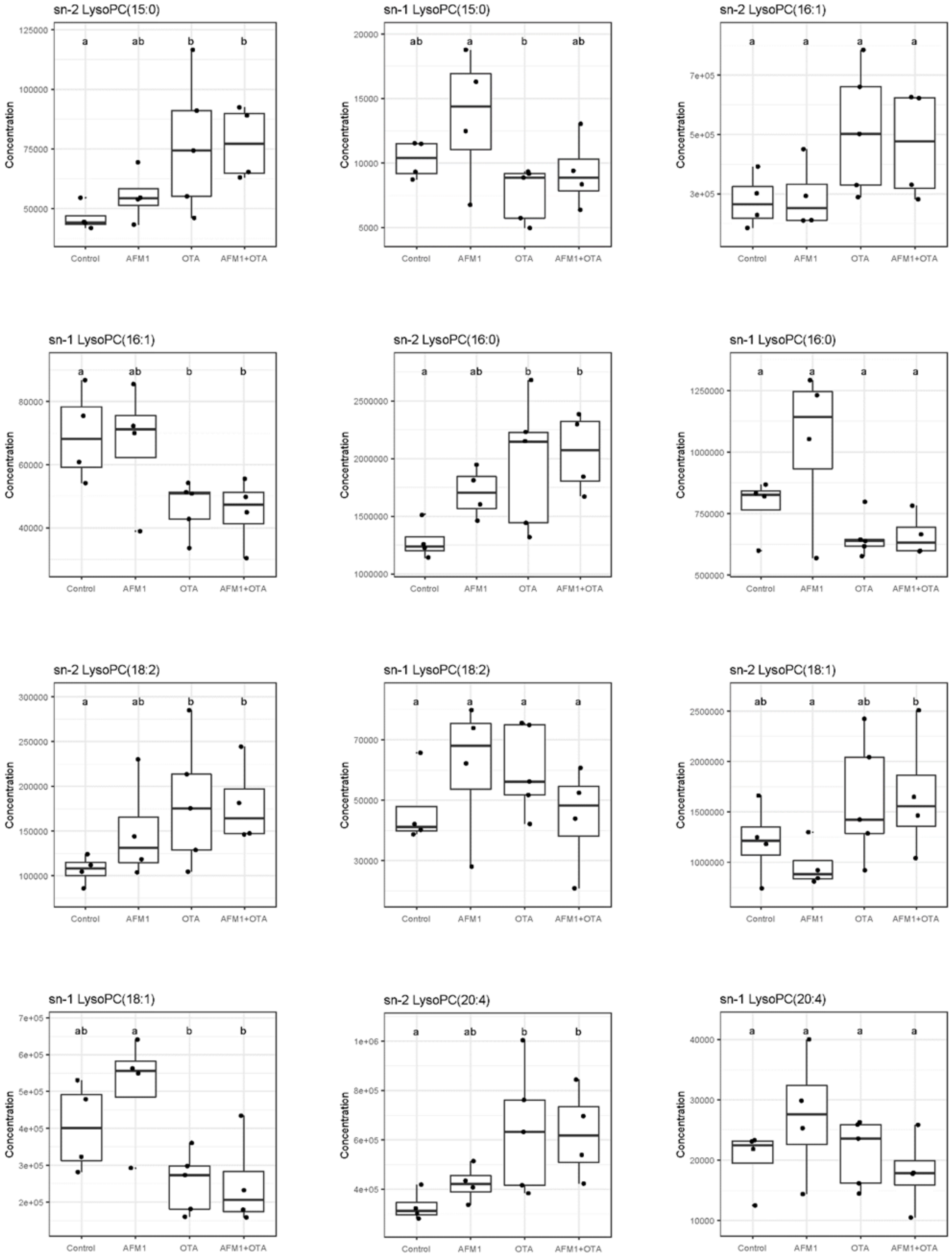
sn-2 LysoPC(15:0)
a ab b b
sn-1 LysoPC(15:0)
ab a b ab
sn-2 LysoPC(16:1)
a a a a
sn-1 LysoPC(16:1)
a ab b b
sn-2 LysoPC(16:0)
a ab b b
sn-1 LysoPC(16:0)
a a a a
sn-2 LysoPC(18:2)
a ab b b
sn-1 LysoPC(18:2)
a a a a
sn-2 LysoPC(18:1)
ab a ab b
sn-1 LysoPC(18:1)
ab a b b
sn-2 LysoPC(20:4)
a ab b b
sn-1 LysoPC(20:4)
a a a a
Concentration
Control
AFM1
OTA
AFM1+OTA

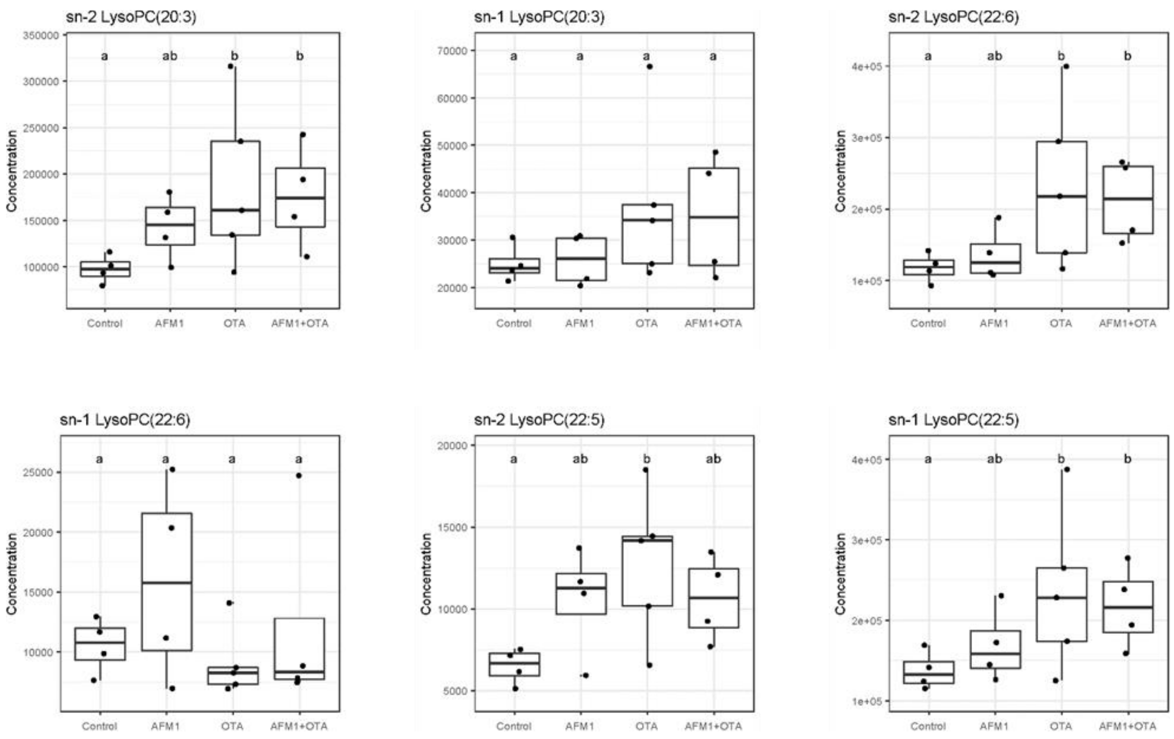

Figure S7. The concentration of significant changed LysoPCs in HepG2 cells.
